# NANOG regulates the proliferation of PCSCs via the TGF-β1/SMAD pathway

**DOI:** 10.1515/med-2020-0221

**Published:** 2020-09-01

**Authors:** Changming Liu, Mingxiong Sheng, Liheng Lin, Huizhang Li, Shanming Guo, Jiabin Zhang, Guangbing Chen, Huihong Chen

**Affiliations:** The Department of Urology, Mindong Hospital Affiliated to Fujian Medical University, Fuan, Fujian 355000, People’s Republic of China

**Keywords:** castration-resistant prostate cancer, prostate cancer stem cells, NANOG, TGF-β1/SMAD signal, proliferation

## Abstract

**Purpose:**

In prostate cancer, castration resistance is a factor that frequently leads to death in individuals with this disease. Recent studies have suggested that prostate cancer stem cells (PCSCs) are pivotal regulators in the establishment of castration resistance. The nanog homeobox (NANOG) and the transforming growth factor (TGF)-β1/drosophila mothers against decapentaplegic protein (SMAD) signaling pathways are involved in several cancer stem cells but are not involved in PCSCs. The purpose of this study is to investigate the effect of NANOG on the proliferation of PCSCs regulated by the TGF-β1/SMAD signaling pathway.

**Methods:**

In this study, we used flow cytometry to isolate CD44+/CD133+/NANOG+ PCSCs from DU145 prostate cancer cells. Then we used short hairpin RNA to silence NANOG and observed the biological behavior and the TGF-β1/SMAD signal of PCSCs.

**Results:**

NANOG decreased PCSC proliferation, increased apoptosis, and blocked cell cycling at G0/G1. Furthermore, reduction in the TGF-β1, p15, and p-SMAD2 expression was observed.

**Conclusion:**

These findings suggest that NANOG positively regulates the growth of PCSCs through the TGF-β1/SMAD signaling pathway.

## 1 Introduction

Prostate cancer is a harmful disease that endangers human health globally. The traditional treatment for prostate cancer is endocrine therapy or castration to avoid androgen stimulation, which can enhance the growth of prostate cancer cells and promote disease development or progression. Endocrine therapy failure can result in the cancer cells gaining castration resistance and continuing to grow. Prostate cancer then develops into castration-resistant prostate cancer, which is the primary cause of death. Studies have shown that there is no clear evidence of prolonged survival regarding endocrine therapy, including withdrawal of anti-male drugs, replacement of anti-male drugs, and switching to estrogen. It is especially important to study the mechanism of castration resistance in prostate cancer and explore a theoretical basis for an innovative and effective treatment.

Prostate cancer stem cells (PCSCs) are suggested to be the most important driver of the development of prostate cancer into castration-resistant prostate cancer. PCSCs are major cells involved in oncogenic transformation and play a critical biological role in the development of prostate cancer. Immortalization is one of the common characteristics of stem cells, and it can increase the chemical resistance of cells against cancer agents [1]. In the presence of castration-resistant stem cell populations in the prostate epithelium, readministration of androgen at physiological levels after castration can promote prostate regeneration. Studies have revealed that exhaustion of cancer stem cells results in a loss of self-renewal in prostate cancer models as well as a loss of tumorigenic capacity [2]. Mutations in phosphatase and tensin homolog (PTEN) and TP53 are commonly present in castration-resistant prostate cancer. They inhibit the basic functions of PCSCs, thereby promoting metastasis and plasticity of prostate cancer cells [3].

Recent studies have shown that the *NANOG* gene is an important tumor stem cell marker involved in cell proliferation, renewal, and pluripotency. It has been found that knocking out NANOG in early or hypoxic cultured mesenchymal stem cells reduces proliferation and differentiation potential and increases spontaneous differentiation. NANOG directly binds to the DNA (cytosine-5-)-methyltransferase 1 (DNMT1) promoter to modulate the self-renewal ability of mesenchymal stem cells [4]. Tcf7I1 attenuates the self-renewal ability of the primary liver cancer stem cells by NANOG-induced transcriptional inhibition [5]. Overexpression of the Raf kinase inhibitory protein or AlkB family member 5, RNA demethylase (ALKBH5) can increase the expression level of NANOG, thereby participating in the regulation of cancer stem cell growth and reducing the capacity for tumor initiation [6,7]. Furthermore, in the case of high expression of NANOG, the histone deacetylase 1 (HDAC1) can be inhibited to reduce the stem cell invasion [8]. However, the role of NANOG in PCSCs is currently unknown.

Recently, it has been proven that the treatment of myeloma stem cells with transforming growth factor (TGF)-β/SMAD signaling inhibitors could reduce stem cell colony formation. Paracrine signaling affects human adipose stem cells or breast cancer stem cells through the cross-talk between the TGF-β/SMAD and the PI3K/serine-threonine kinase (AKT) pathways [9,10]. The TGF-β/SMAD pathway can also induce the formation of breast cancer tumor-initiating cells [11]. In addition, the microRNA, miR-106b family, regulates cancer stem cell self-renewal and decreases cell invasion by downregulating the TGF-β/SMAD signaling pathway [12]. The TGF-β/SMAD pathway is vital in many cancer stem cells. Furthermore, the *NANOG* gene is suggested to regulate the expression of the TGF-β/SMAD pathway in the KEGG database. However, there is a lack of knowledge on the role of NANOG in the TGF-β/SMAD pathway in PCSCs.

This study knocked down NANOG by small interfering RNA (siRNA) in PCSCs and investigated the biological behavior of PCSCs and the expression of the TGF-β/SMAD signaling pathway to explore the possible regulatory mechanism of NANOG in PCSCs.

## 2 Materials and methods

### 2.1 Isolated PCSCs

Human prostate cancer DU145 cell lines were purchased from the American Type Culture Collection (ATCC, Manassas, VA, USA). The DU145 cells were grown in Roswell Park Memorial Institute-1640 medium. The medium was supplemented with 10% fetal bovine serum, 4 mL glutamine, 100 U/mL penicillin, and 100 µg/mL streptomycin. The cells were incubated at 37°C in a humidified atmosphere of 5% CO_2_.

The DU145 cells were counted and resuspended in the flow buffer (1 × 10^7^ cells were resuspended in 500 µL of buffer). Flow buffer: PBS, pH = 7.2, 0.5% BSA, and 2 mol ethylenediaminetetraacetic acid were added. The cells were incubated with anti-CD133 (eBioscience, California, USA), anti-CD44 (eBioscience), and anti-NANOG (Invitrogen, California, USA) antibodies at 4°C for 30 min in the dark. The cells were then resuspended in 500 µL flow buffer. The CD44+/CD133+/NANOG+ PCSCs were sorted by flow cytometry (Beckman MoFloXDP). PCSCs were cultured in a complete growth medium supplemented with 10% serum.

### 2.2 siRNA interference

The PCSCs were passaged 16 h before transfection, and the cells grew to 80% confluence before transfection. The NANOG-siRNA and scramble-siRNA were then transfected according to the procedures outlined in the Lipofectamine 2000 reagent instructions. The cells were harvested 8 h after transfection for Western blot and analyzed to identify the interference effects of NANOG-siRNA in comparison with scramble (R) siRNA-transfected cells. The sequence of siRNA is as follows: short hairpin RNA (shRNA1F): GATCCGATAG ATTTCAGAGACAGATTCAAGAGATCTGTCTCTGAAATCTATCTTTTTG, shRNA1R: AATTCAAAAAGATAGATTTCAGAGACAGATCTCTTGAATCTGTCT CTGAAATCTATCG, shRNA NC F: GATCCCAGAGATTTTCAGAGAAGATTCAA GAGATCTTCTCTGAAAATCTCTGTTTTTG, shRNA NC R: AATTCAAAAACAG AGATTTTCAGAGAAGATCTCTTGAATCTTCTCTGAAAATCTCTGG.

### 2.3 Cell cycle detection

Cell cycle assays were performed using Annexin propidium iodide (PI) staining. Each group of cells was inoculated into a six-well plate, and the complete culture solution was replaced according to the state of the cells. The cells were cultured for 48 h, stored in 70% alcohol at 4°C for 24 h, and then stained for 30 min in the dark. The specific steps are as follows: first, 1 to 5 × 10^5^ cells were harvested and washed twice with pre-cooled PBS (2,000 rpm, 5 min to collect cells). Second, pre-cooled 70% ethanol was added. Third, the cells were fixed at 4°C overnight. The cells were resuspended in 500 µL of 1× binding buffer in advance and 50 µL of PI was added (according to the instruction 50:1). This solution was gently mixed and then finally analyzed by flow cytometry. Approximately 2–3 million cells were counted, and the results were analyzed by ModFit. During analysis, the cells were removed using FL2-w and FL2-A. The stained cells were tested within 1 h, as much as possible.

### 2.4 MTT assay for cell proliferation

The PCSCs were seeded (1 × 10^4^ cells/well, 96 well plates). After 16 h of culture, 3-Deazaneplanocin A (DZNep) was added (the final concentrations were 0, 0.25, 0.5, 1, 2, 4, and 8); 16 µmol/L continued to culture for 12, 24, and 48 h. In the experiment, an equal volume of DMSO (DZNep solvent, concentration 0.01% DMSO) was used as a control, and three duplicate wells were set for each drug concentration. One hundred microliter of basal medium containing MTT was added after the specified time point to ensure a final MTT concentration of 200 µg/mL. Two hundred microliter of DMSO was added to each well and shook at room temperature for 15–20 min to completely dissolve the crystals. The OD value was measured at a wavelength of 490 nm using an enzyme labeling instrument.

### 2.5 Western blot

The treatment group and its control group PCSCs were treated with RIPA whole cell lysate at a predetermined time point. After lysis on ice for 15 min, 10,000 *g* were centrifuged for 10 min and the liquid was collected. The total protein content in the supernatant of the obtained cell lysate was quantified using the bicinchoninic acid (BCA) method. It was then separated by 10% SDS-PAGE (25 µg/well) and electroporated to the NC membrane (BioTrace, Pall, USA). The membrane was blocked with a solution of tris buffered saline tween (TBST) (20 mmol/L of Tris–HCl, 150 mmol/L of NaCl, 0.05% Tween-20, pH 7.4) containing 5% skim milk for 1 h at room temperature, and specific primary antibody diluted with 5% skim milk (TBST) incubated overnight at 4°C and then peroxidized with horseradish. The enzyme-labeled secondary antibody (1:3,000 diluted in TBST) was incubated for 1 h at room temperature. Finally, the electrogenerated chemiluminescence (ECL) reagent was developed. In the experiment, β-actin was used as an internal reference protein.

### 2.6 Statistical analysis

The data obtained from the experiment are shown by mean ± standard deviation. The mean comparison between groups was analyzed using a two-sided *t* test (SPSS 17.0 statistical analysis software). One-way ANOVA were performed with statistical significance as follows: *p < 0.05, **p < 0.01, and ***p < 0.001.

## 3 Results

### 3.1 shRNA reduced the expression of NANOG in PCSCs

**Figure 1:**
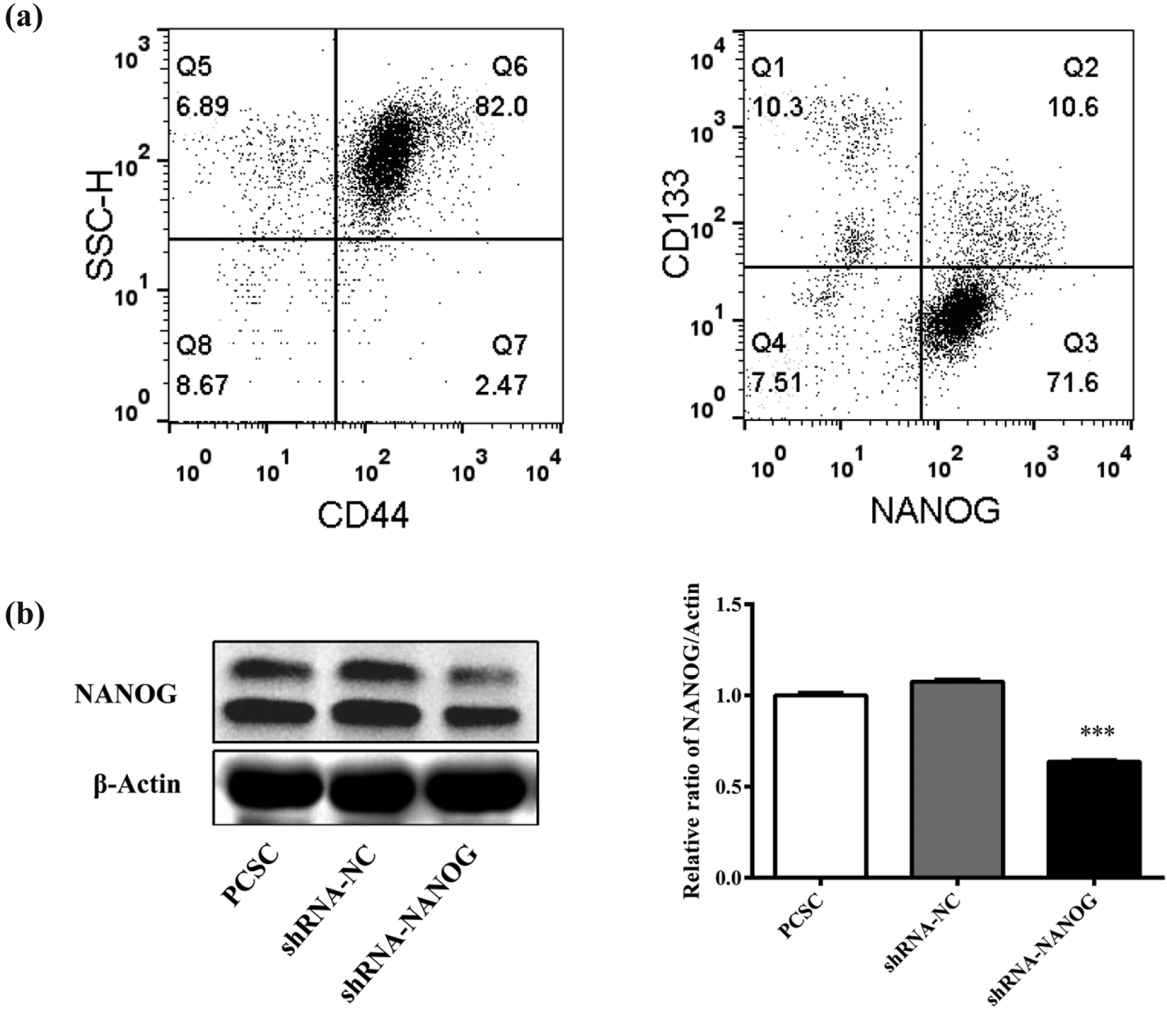
shRNA downregulates the expression of NANOG in PCSCs. Screening for CD44/CD133/NANOG-positive PCSC by flow cytometry (a). Western blot was used to detect the level of protein expression of NANOG (b). After the transfection of sh-NANOG, the expression of NANOG was significantly downregulated (*p < 0.05, **p < 0.01, and ***p < 0.001).

First, flow cytometry was performed to selecte CD44/CD133/NANOG-positive PCSCs. From the resuts, PCSCs accounted for 8.97% (Figure 1a). The protein level of NANOG was detected using Western blot analysis, and it was found that shRNA-NANOG reduced the amount of protein by half in PCSCs (Figure 1b). NANOG was highly expressed in PCSCs and PCSCs + shRNA-NC, and NANOG was significantly downregulated in PCSCs + shRNA-NANOG (*p* < 0.001).

### 3.2 shRNA-NANOG inhibits PCSC proliferation

**Figure 2:**
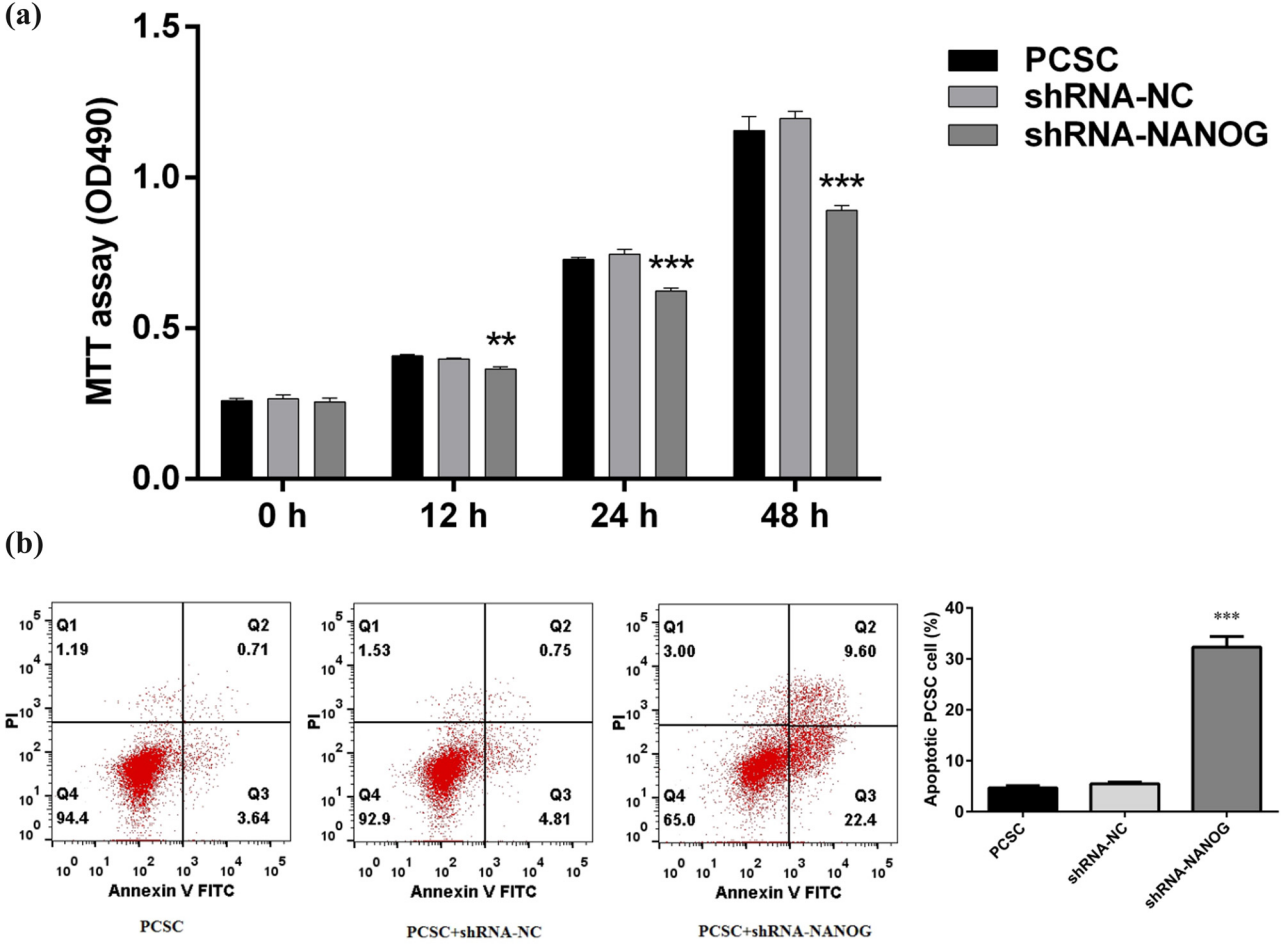
shRNA-NANOG inhibits PCSC proliferation and promotes PCSC apoptosis. (a) MTT assay was used to detect the proliferation of PCSC within 48 h of shRNA-NANOG transfection. After 12 h of shRNA-NANOG treatment, cell proliferation ability was significantly downregulated (*p < 0.05, **p < 0.01, and ***p < 0.001), and the proliferation ability of cells was significantly inhibited after 24 and 48 h (*p < 0.05, **p < 0.01, and ***p < 0.001). (b and c) Flow cytometry was used to detect apoptosis. shRNA-NANOG significantly promoted apoptosis of PCSCs (*p < 0.05, **p < 0.01, and ***p < 0.001).

After reducing the level of NANOG in PCSCs, cell proliferation was analyzed by MTT assay (Figure 2a). At 24 and 48 h, the number of cells in the shRNA-NANOG group was significantly lower than that of the control group (*p* < 0.001). PCSCs + shRNA-NANOG had significantly inhibited proliferation compared to PCSCs and PCSCs + shRNA-NC.

### 3.3 shRNA-NANOG promotes apoptosis of PCSCs

The proportion of apoptosis was investigated after reducing the level of NANOG in PCSCs (Figure 2b). The results showed that the percentage of apoptotic PCSCs in the control group was approximately 5%, while the percentage in the shRNA-NANOG group increased to 30% (*p* < 0.001). Compared to PCSCs and PCSCs + shRNA-NC, PCSCs + shRNA-NANOG had significantly inhibited proliferation and increased apoptosis.

### 3.4 PCSC cycle is blocked by shRNA-NANOG

**Figure 3:**
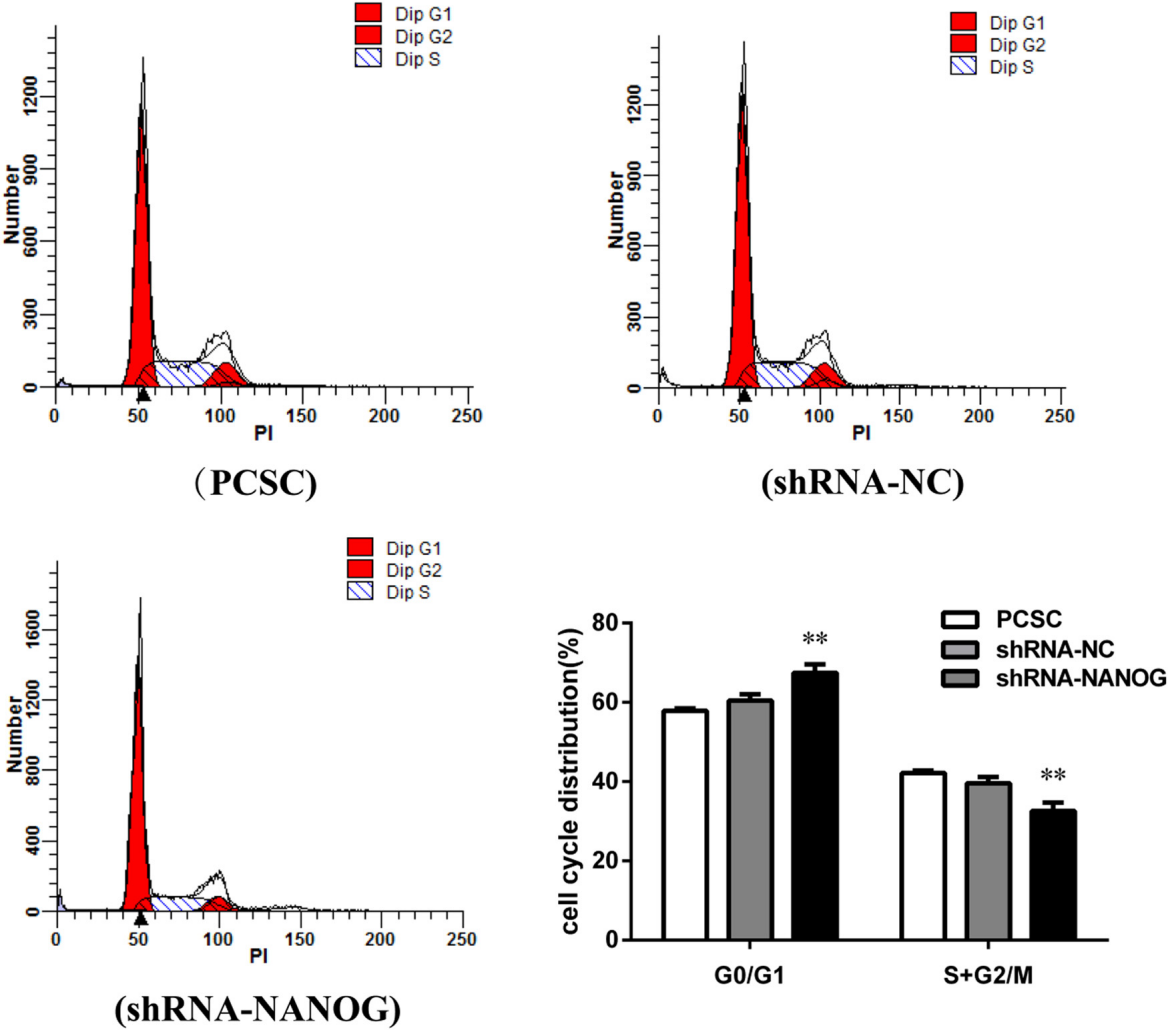
Interfering with NANOG in PCSCs blocks the cell cycle. (a and b) After the transfection of PCSCs with shRNA-NANOG, flow cytometry was performed to detect cell cycle conditions. The proportion of the G0/G1-phase cells and the S + G2/M-phase cells is significantly upregulated (*p < 0.05, **p < 0.01, and ***p < 0.001) and significantly downregulated (*p < 0.05, **p < 0.01, and ***p < 0.001), respectively.

After transfecting PCSCs with shRNA-NC and shRNA-NANOG, the cell cycle distribution was investigated (Figure 3). The results showed that the proportion of the G0/G1-phase and S + G2/M-phase cells in the control group was 60% and 40%, respectively (*p* < 0.01). In the shRNA-NANOG group, these proportions were approximately 70% and 30% (*p* < 0.01). Compared to PCSCs and PCSCs + shRNA-NC, PCSCs + shRNA-NANOG were more often arrested in G0/G1 phase, and the proportion of S + G2/M phase cells significantly decreased.

### 3.5 shRNA-NANOG regulates PCSCs via TGF-β/SMAD signaling pathway

**Figure 4:**
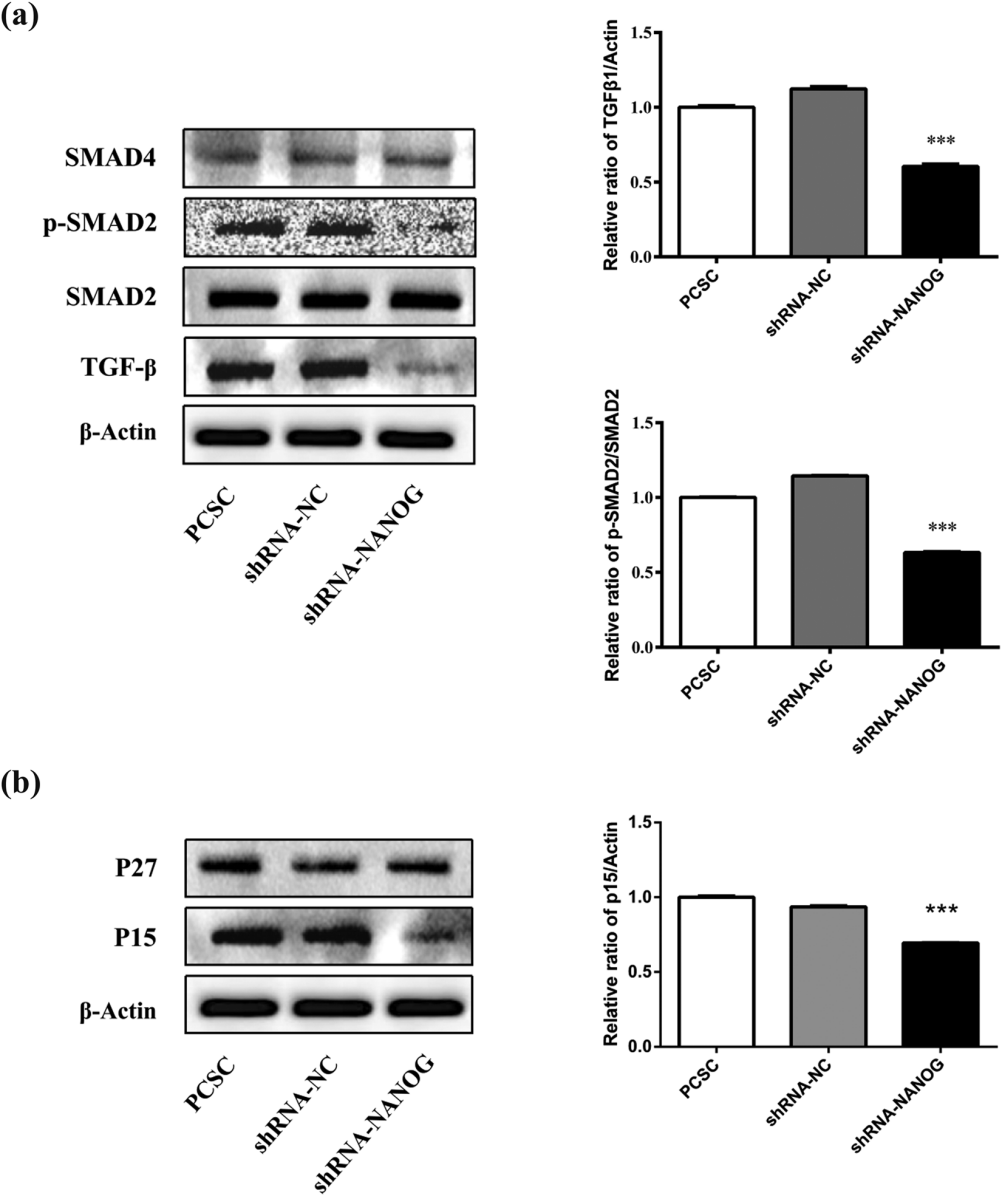
shRNA-NANOG regulates the TGF-β1/SMAD signaling pathway. The protein levels of TGF-β1, SMAD2, p-SMAD2, SMAD4, p27, and p15 were detected using Western blot analysis. The inhibition of NANOG significantly downregulated the protein expressions of TGFβ1, p15, and p-SMAD2 (*p < 0.05, **p < 0.01, and ***p < 0.001), while it had little effect on p27, SMAD4, and SMAD2.

The TGF-β/SMAD pathway may be a downstream signaling pathway of NANOG in PCSCs. We examined the expression levels of proteins in this pathway (Figure 4). The results showed that the TGF-β1 expression decreased in PCSCs + shRNA-NANOG compared to PCSCs and PCSCs + shRNA-NC. shRNA-NANOG did not affect the PCSC expression of SMAD2 or SMAD4 proteins but downregulated p-SMAD2 (significantly, *p* < 0.01), p27 (not significantly), and p15 levels (significantly, *p* < 0.01).

### 3.6 TGF-β1 rescued the proliferation inhibition induced by shRNA-NANOG

**Figure 5:**
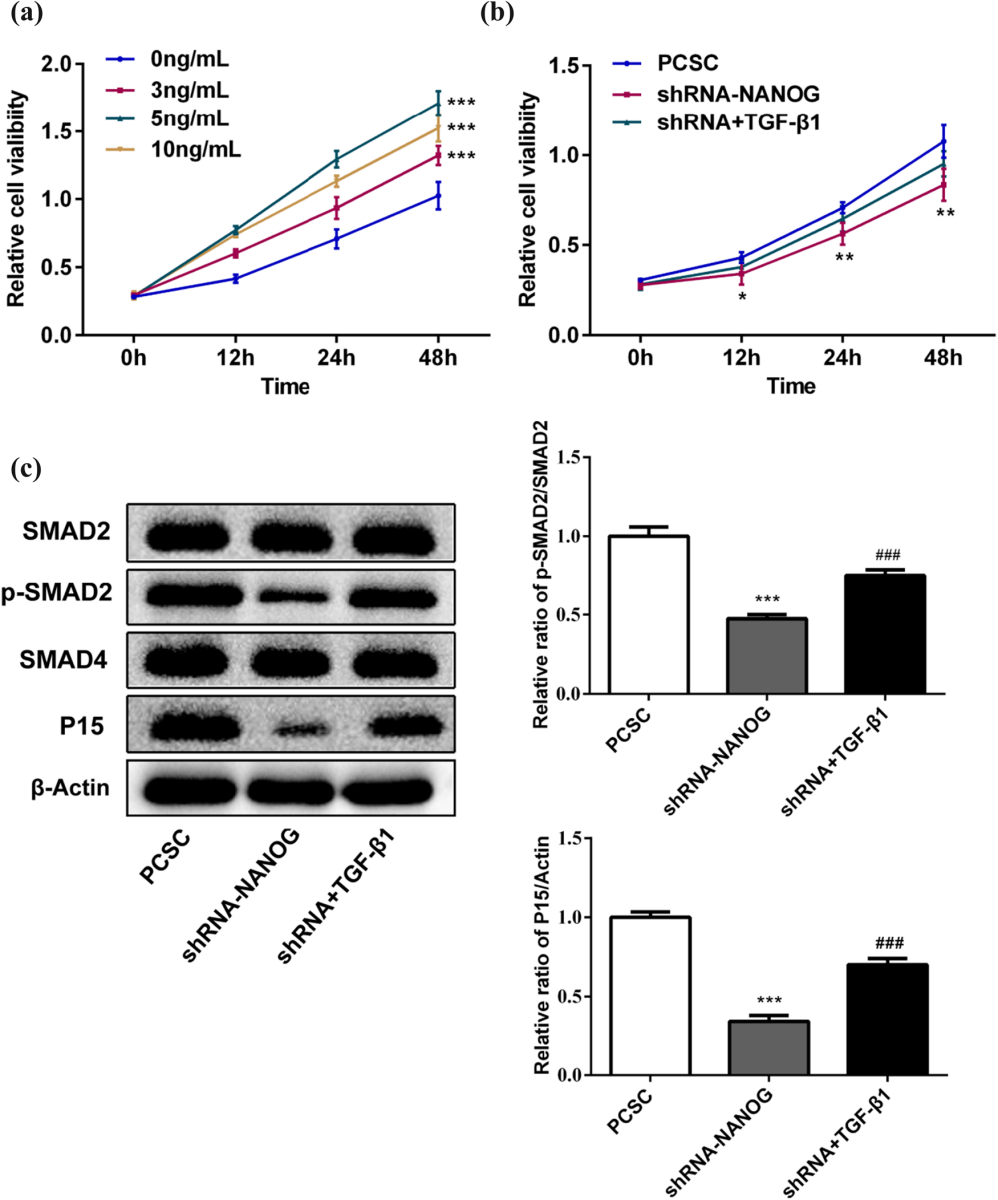
TGF-β1 rescued the proliferation inhibition induced by shRNA-NANOG. (a) The effect of TGF-β1 on PCSC proliferation was detected by MTT assay. ***p < 0.001 compared to the 0 ng/mL group. (b) The effect of TGF-β1 on the proliferation inhibition induced by shRNA-NANOG was detected by MTT assay. *p < 0.05 and **p < 0.01 compared to the PCSC group. (c) TGF-β1 intervention increases the expression of p-SMAD2 and P15 in shRNA-NANOG cells. ***p < 0.001 with the PCSC group; ^###^ *p* < 0.001 compared to the shRNA-NANOG group.

Finally, we treated the PCSCs with TGF-β1 (0, 3, 5, and 10 ng/mL). As shown in Figure 5a, TGF-β1 promoted the proliferation of PCSCs, and 5 ng/mL of TGF-β1 had the best effect on PCSCs. The NANOG knockdown cells were treated with 5 ng/mL TGF-β1. Compared to the control group, the proliferation of PCSCs significantly increased after TGF-β1 treatment (Figure 5b). In addition, TGF-β1 also rescued the inhibition of SMAD2 phosphorylation and P15 expression induced by shRNA-NANOG (Figure 5c). These data proved that TGF-β1 could block the effect of shRNA-NANOG on PCSCs.

## 4 Discussion

Most prostate cancer patients eventually develop castration resistance within 2 years after castration. At present, the pathogenesis of castration-resistant prostate cancer is roughly divided into two categories: adaptation mechanisms and selection mechanisms. Adaptation mechanisms include prostate cancer cell gene mutations, androgen receptor mutations, and androgen receptor gene overexpression. Selection mechanisms include castration-resistant subcloning, which is supported by the preexisting theory and cancer stem cell theory. The cancer stem cells have the following characteristics: unlimited self-renewal, differentiation potential, heterogeneity, self-protection, and high tumorigenicity. The cancer stem cells may have an important function in tumorigenesis, development, distant metastasis, and therapeutic resistance of cancer.

In this study, it was found that NANOG participates in the regulation of PCSC proliferation, apoptosis, and the cell cycle. It can promote PCSC proliferation, cell cycle and inhibit apoptosis. NANOG has been shown to be expressed in a variety of human tumors and plays an important role in tumors. The inhibition of NANOG expression can decrease the proliferation of breast cancer stem cells and esophageal cancer stem cells, thereby impeding the formation of cancer cell colonies [13,14]. Human NANOG pseudogene 8 was found to participate in the development of tumors in a subset of gastric cancer patients, by affecting the proliferation of cancer stem cells in the digestive tract [15]. Furthermore, NANOG interacts with androgen receptors to promote the proliferation and migration of ovarian cancer stem cells [16]. The IGF2/IGF1R/NANOG signaling pathway has important functions in leukemia stem cell proliferation, and the knockdown of NANOG induces cell cycle arrest and apoptosis [17]. In conclusion, the role of NANOG in PCSCs is the same as in other cancers, i.e., controlling cancer stem cell growth.

This study found that silencing NANOG decreased the expressions of TGF-β1 and p15 and reduced the phosphorylation of SMAD2. The SMAD protein is a signal transduction and transcriptional regulator recruited by the TGF-β receptor, mediating the signal of TGF-β, thereby regulating a variety of biological processes. These include cell proliferation, apoptosis, and differentiation [18,19,20,21]. Evidence suggests that SMAD family member 1 plays an integral role in different cancer types, such as lung cancer and colorectal cancer [22,23]. SMAD4 also plays a role in cancer development, including colorectal liver metastases and pancreatic cancer [24,25]. Furthermore, SMAD signaling can induce epithelial−mesenchymal transition in colorectal cancer [26], pancreatic cancer [27], and breast cancer [10]. It can also promote tumor cell metastasis and invasion. Interferon gamma induces DNA damage and promotes senescence of cancer cells through TGF-β/SMAD signaling [28]. In general, the function of NANOG in regulating the PCSC growth may be achieved through the TGF-β/SMAD signaling pathway.

In conclusion, we found that silencing NANOG can decrease the expression and phosphorylation of TGF-β/SMAD signaling pathway components, inhibit proliferation of PCSCs, promote apoptosis, and arrest the cell cycle. NANOG is a key factor in regulating the growth of PCSCs and is a possible target for the treatment of prostate cancer. Our conclusions may assist in the development of new treatments of castration-resistant prostate cancer and radiotherapy.
